# Chlorogenic acid alleviates obesity and modulates gut microbiota in high‐fat‐fed mice

**DOI:** 10.1002/fsn3.868

**Published:** 2019-01-28

**Authors:** Zhengyu Wang, Ka‐Lung Lam, Jiamiao Hu, Shenghan Ge, Arong Zhou, Baodong Zheng, Shaoxiao Zeng, Shaoling Lin

**Affiliations:** ^1^ College of Food Science Fujian Agriculture and Forestry University Fuzhou China; ^2^ Fujian Provincial Key Laboratory of Quality Science and Processing Technology in Special Starch Fujian Agriculture and Forestry University Fuzhou China; ^3^ School of Life Sciences The Chinese University of Hong Kong Shatin Hong Kong S.A.R. China

**Keywords:** body weight, chlorogenic acid, gut microbiota, obesity

## Abstract

To evaluate the anti‐obesity effects of chlorogenic acid (CGA), the mice were fed a high‐fat diet (HFD) upon chlorogenic acid treatment for 6 weeks. The results showed administration of chlorogenic acid (150 mg per kg per day) remarkably promoted body loss, reduced lipid levels in plasma and altered mRNA expression of lipogenesis and lipolysis related genes in adipose tissue. Moreover, chlorogenic acid also reversed the HFD‐induced gut microbiota dysbiosis, including significantly inhibiting the growth of *Desulfovibrionaceae, Ruminococcaceae, Lachnospiraceae, Erysipelotrichaceae,* and raising the growth of *Bacteroidaceae, Lactobacillaceae*. Overall, the amelioration of HFD‐induced gut microbiota dysbiosis by chlorogenic acid may contribute, at least partially, to its beneficial effects on ameliorating HFD‐induced obesity.

## 1 INTRODUCTION

Obesity has become a serious concern because it is tightly associated with cardiovascular morbidity, inflammations, type 2 diabetes and etc. via complex interrelationships with unfavorable metabolic consequence (Hu, Lin, Zheng, & Cheung, 2018; Kobayashi, Kawasaki, Takahashi, Maeno, & Nomura, 2017). At present, the researchers have drawn wide attention on the relationship between host gene (gene‐induced obesity), dietary excesses (diet‐induced obesity), and obesity (Wei, Li, Zhao, & Nicholson, 2008). In fact, alteration of intestinal flora was also identified as an important element in close association with the obesity and obesity‐induced metabolic disorders (Eaimworawuthikul, Thiennimitr, Chattipakorn, & Chattipakorn, 2017; Shen et al., 2017). The reduced *Firmicutes*/*Bacteroidetes* ratio has been associated with improved glucose levels, body weight, and fat reduction (Kemperman et al., 2013; Singh et al., 2017). In brief, intestinal flora is indispensable to maintain gut homeostasis and, in turn, control the obesity and obesity‐related diseases.

Plant‐based foods are rich in source of antioxidant phenolics (Zhao, Kao et al., 2017). The polyphenols including quercetin (Zheng et al., 2017), puerarin (Xue et al., 2016), catechin (Huang et al., 2016) have been reported to ameliorate host obesity associated with a high‐fat diet by modulating gut microbiota. Indeed, as potential prebiotic agents, chlorogenic acid has been found to exhibit anti‐obesity property, especially improve lipid and glucose metabolism (Cho et al., 2010; Lin, Hu, Zhou, & Cheung, 2017; Sotillo & Hadley, 2002). Besides, evidence also supports chlorogenic acid (100 μg/mL) can significantly increase the abundance of *Firmicutes, Bacteroides* in vitro (*p *<* *0.01) (Parkar, Trower, & Stevenson, 2013). Therefore, we hypothesized that chlorogenic acid may also influence the gut microbiota in vivo, which might be one of the underlying mechanisms by which chlorogenic acid exerts its anti‐obesity effects. To test our hypothesis, we assessed the potential of chlorogenic acid in altering the gut microbiota composition as well as maintaining gut homeostasis upon HFD challenge in this study. Also, combining physiological and biochemical parameters, and relative genes expression to demonstrated chlorogenic acid is useful for the prevention and treatment of obesity.

## 2 METHODS AND MATERIALS

### 2.1 Animals and diets

Eighteen ICR male mice (clean grade, the Wu laboratory animal trading co., Ltd., Fuzhou, China), 5‐to 6‐weeks old, with body weights ranging from 29 to 31 g. Mice were housed up to six per cage with a 12‐hr light/12‐hr dark cycle (lights on from 8:00 a.m.to 8:00 p.m.) at 23 ± 1°C and 50 ± 10% humidity. All mice were fed a normal diet and adapt environmental one week. They were then randomly separated into three groups: normal diet group (ND), high‐fat diet model group (HFD), and high‐fat diet with 150 mg/kg bw/day of chlorogenic acid (CGA). The normal diet contained (in weight percent): 22.3% protein, 60.6% carbohydrate, and 4.0% fat, high‐fat diet contained: 21.6% protein, 43.1% carbohydrate, and 18.4% fat. All mice were treated orally by gavage, the ND and HFD groups received an oral saline, and CGA group received chlorogenic acid dissolved in saline at the same volume. The food and water were available ad libitum during 6‐week administration. Body weight was measured every three days at a time.

### 2.2 Biochemical analysis

After 6 weeks of feeding, all animals were fasted 12 hr and weighed. The blood was collected by EDTA tubes and centrifuged at 1500 *g* for 10 min at 4°C. The levels of triglyceride (TG), total cholesterol (TC), high‐density lipoprotein cholesterol (HDL‐C), low‐density lipoprotein cholesterol (LDL‐C), aspartate transaminase (AST), alanine transaminase (ALT), and blood urea nitrogen (BUN) in plasma were evaluated by using automatic biochemical analyzer (7080; Hitachi Co., Japan).

### 2.3 Histological analysis

Epididymal white adipose tissues (WAT) and livers were fixed with 4% paraformaldehyde for 24 hr and embedded in paraffin. Then, 5‐μm sections were prepared and stained with hematoxylin and eosin (H&E). The physiology of epididymal WAT and livers were observed by inverted microscope (Motic BA210T, China).

### 2.4 Quantitative RT‐PCR

Total RNA was extracted from epididymal WAT using an Uniq‐10 Trizol total RNA extraction kit (Sanggon Biotech Co., Ltd., Shanghai, China). cDNA was synthesized with 0.8 μg of total RNA by RevertAid First strand cDNA Synthesis kit. RT‐PCR was performed using the SYBR Green Abstract PCR Mix (Sanggon Biotech Co., Ltd., Shanghai, China) and LightCycler 480 II system (Roche, Basel, Switzerland). The mRNA levels of target genes were normalized to β‐actin. Primer sequences are shown in Supporting Information Table S1.

### 2.5 Gut DNA extraction

The luminal contents of the cecum were isolated to extract the total bacterial community DNA using the DNeasy PowerSoil Kit (QIAGEN, Inc., Netherlands), following the manufacturer's instructions, and stored at −20°C prior to further analysis. The quantity and quality of extracted DNAs were measured using a NanoDrop ND‐1000 spectrophotometer (Thermo Fisher Scientific, Waltham, MA, USA) and agarose gel electrophoresis, respectively.

### 2.6 Illumina high‐throughput sequencing of barcoded 16S rRNA genes

PCR amplification of the bacterial 16S rRNA genes V3–V4 region was performed using the forward primer 338F(5′‐barcode+ACTCCTACGGGAGGCAGCA‐3′) and the reverse primer 806R (5′‐GGACTACHVGGGTWTCTAAT‐3′). Sample‐specific 7‐bp barcodes were incorporated into the primers for multiplex sequencing. After that, the 16s rDNA sequencing and analysis were performed as described previously (Yang, Dou, & An, 2018).

### 2.7 Data analysis and statistics

All data were presented as the mean value ± standard deviation (*SD*) and comparisons of data were carried out using a Student's *t* test or a one‐way analysis of variance (ANOVA) with Duncan's test. Values of *p *<* *0.05 were considered to be statistically significant.

## 3 RESULTS

### 3.1 Chlorogenic acid reduced HFD‐induced body weight and fat weight increase

**Figure 1:**
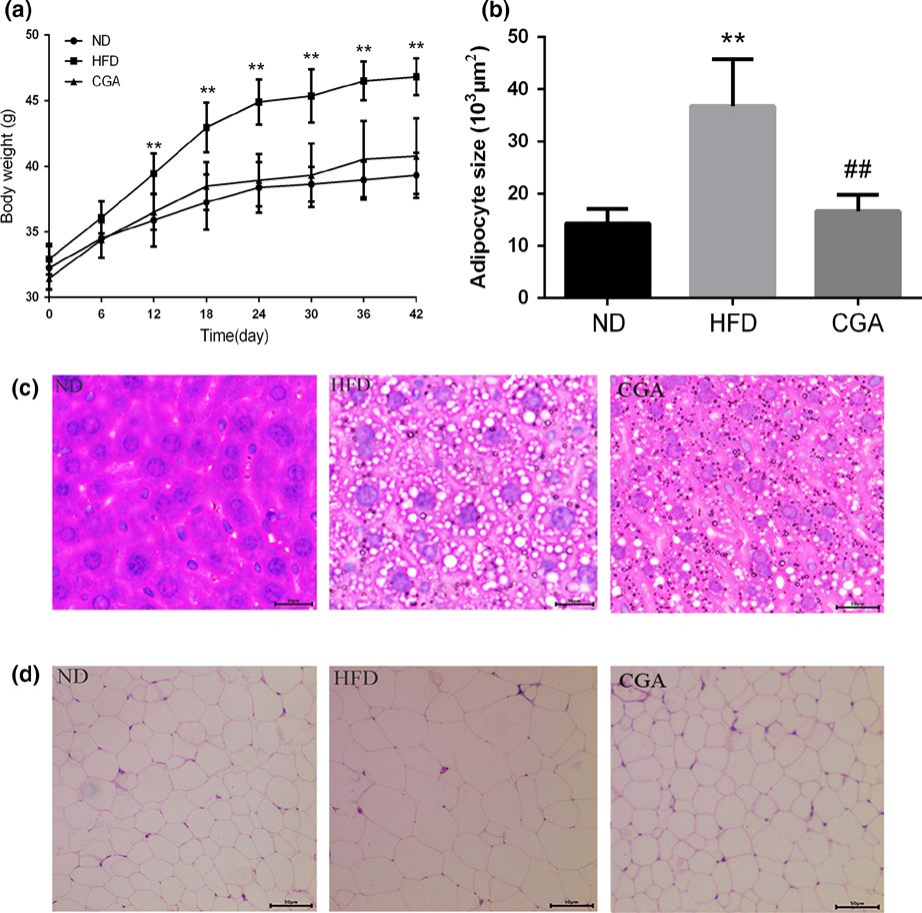
Effects of chlorogenic acid on body weight increase of mice during the 6‐week study period (a). The sizes of adipocytes in epididymal adipose tissue (b). Liver morphology (c) and epididymal WAT morphology (d) in different groups. HE staining (×200). Significant differences between HFD versus ND are indicated: *p <* *0.05; **p <* *0.01. Significant differences between CGA versus HFD are indicated: ^#^ *p *<* *0.05; ^##^ *p *<* *0.01

The body weight of mice in CGA group changed slowly during a 6‐week feeding period (Figure 1a), and the mice fed HFD supplemented with chlorogenic acid (150 mg/kg) had significantly lower body weight in comparison to HFD‐fed mice (Table 1). Further, the weight gain, liver weight, fat weight were also markedly decreased by chlorogenic acid treatment (*p < *0.05). Histological section from epididymal WAT indicated fat mass and adipocyte size were greatly attenuated by CGA administration (Figure 1b,d). Similarly, liver histological section in HFD‐fed mice also showed abnormal hepatic steatosis and large amounts of lipid droplets, but chlorogenic acid can diminish these adverse changes (Figure 1c). Thus, these results confirmed that chlorogenic acid has anti‐obesity effects.

**Table 1:** Effects of chlorogenic acid on body measurements in different groups at the end of 6‐week HFD feeding

Groups	ND	HFD	CGA
Initial bodyweight (g)	32.25 ± 1.65^a^	32.93 ± 1.17^a^	31.53 ± 0.86^a^
End body weight (g)	39.31 ± 1.71^c^	46.82 ± 1.40^a^	40.79 ± 1.07^b^
Body weight gain (g)	6.91 ± 1.69^c^	13.90 ± 1.57^a^	9.33 ± 2.14^b^
Liver weight (g)	1.46 ± 0.07^c^	1.82 ± 0.20^a^	1.50 ± 0.13^b^
Epididymal WAT weight (g)	0.66 ± 0.11^c^	2.02 ± 0.39^a^	1.18 ± 0.26^b^

*Notes*

All data in the table are mean ± *SD*.

HFD: high‐fat diet; ND: normal diet; CGA: chlorogenic acid; WAT: white adipose tissues.

Values in a row with different superscript letters are significantly different *p *<* *0.05.

### 3.2 Chlorogenic acid improved lipid profile and reduced toxicity in plasma

To examine the dyslipidemia‐preventing effect of chlorogenic acid in HFD‐fed mice, plasma lipid levels were analyzed. Table 2 shows the plasma biochemical variables in mice after 6 weeks of treatment with chlorogenic acid. The TG, TC, LDL‐C levels in the HFD group were significantly increased compared to that in the ND group. However, treatment with chlorogenic acid significantly reduced TC, TG, LDL‐C levels and increased HDL‐C level as compared with the HFD group. Moreover, the hepatic toxicity and renal toxicity were investigated by measuring plasma AST, ALT, and BUN levels, respectively. ALT, AST, BUN levels increased in the HFD group, but chlorogenic acid administration significantly decreased AST and BUN levels compared to the HFD group (*p *<* *0.05).

**Table 2:** Effects of CGA on plasma biochemical indicators in different groups at the end of 6‐week feeding

Groups	ND	HFD	CGA
TG (mmol/L)	1.37 ± 0.28^b^	1.82 ± 0.15^a^	1.39 ± 0.20^b^
TC (mmol/L)	4.09 ± 0.50^c^	5.78 ± 0.75^a^	5.19 ± 0.62^b^
LDL‐C (mmol/L)	0.34 ± 0.10^b^	0.55 ± 0.10^a^	0.43 ± 0.07^b^
HDL‐C (mmol/L)	1.92 ± 0.06^b^	2.10 ± 0.09^b^	2.48 ± 0.27^a^
LDL‐C/HDL‐C	0.19 ± 0.01^b^	0.29 ± 0.04^a^	0.18 ± 0.01^b^
ALT (U/L)	32.50 ± 7.14^a^	45.00 ± 9.63^a^	35.75 ± 4.79^a^
AST (U/L)	119.00 ± 8.16^b^	140.00 ± 14.14^a^	118.40 ± 17.61^b^
BUN (mmol/L)	8.11 ± 0.53^ab^	8.41 ± 0.92^a^	7.52 ± 1.01^b^

*Notes*

All data in the table are mean ± *SD*.

HFD: high‐fat diet; ND: normal diet; CGA: chlorogenic acid; TG: triglyceride; TC: total cholesterol; HDL‐C: high‐density lipoprotein cholesterol; LDL‐C: low‐density lipoprotein cholesterol; AST: aspartate transaminase; ALT: alanine transaminase; BUN: blood urea nitrogen.

Values in a row with different superscript letters are significantly different *p *<* *0.05.

### 3.3 Effects of chlorogenic acid on transcription of genes involved in lipid metabolism

**Figure 2:**
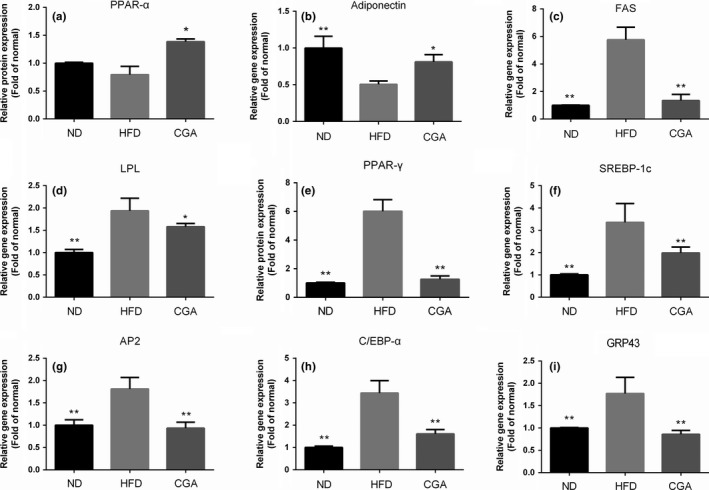
Effect of CGA on mRNA expression of lipid metabolism‐related genes in epididymal adipose tissue. The values of genes levels include peroxisome proliferator‐activated receptor α (PPAR‐α) (a), adponectin (b), FAS (c), LPL (d), PPAR‐γ (e), SREBP‐1c (f), AP2 (g), C/EBP‐α (h), and G protein‐coupled receptor 43(GPR43) (i) were normalized to the value of β‐actin, which was set to 1. *p <* *0.05, **p <* *0.01 compared with the HFD group by the student's *t* test

Based on qPCR results in Figure 2, the mRNA expression of adipocyte markers, such as fatty acid synthase (FAS), lipoprotein lipase (LPL), peroxisome proliferator‐activated receptor γ (PPAR‐γ), sterol regulatory element‐binding protein‐1c (SREBP‐1c), adipocyte protein 2 (AP2), CCAAT/enhancer‐binding protein α (C/EBP‐α), and GRP43 (Figure 2c–i) were remarkably up‐regulated and while mRNA levels of PPARα, adiponectin (Figure 2a,b) were decreased in the epididymal WAT of the HFD mice relative to the ND mice. In contrast, consumption of CGA obviously offset these changes since the tissues of the CGA mice showed significantly lower mRNA expression of PPAR‐γ, AP2, LPL, C/EBPα, FAS, SREBP‐1c and GRP43, and significantly higher mRNA level of PPARα and adiponectin compared to the HFD mice.

### 3.4 Chlorogenic acid modulated gut microbiota at different taxonomic levels

High‐throughput sequencing was applied to explore the effect of chlorogenic acid treatment on the richness and diversity of the gut microbiota. As shown in Table 3, a total of 359,505 validated sequences reads (39,188 for ND group, 48,674 for HFD group, and 41,461 for CGA group) of V3–V4 16S rRNA sequences reads were obtained. As expected in alpha diversity, high‐fat diet intake remarkably decreased diversity of gut microbiota in terms of Shannon indice compared with normal diet consumption (*p *<* *0.01). Noticeably, as *p *>* *0.05 for all indices, the microbial richness and diversity in the chlorogenic acid administration group decreased slightly, but there was no statistical significance between the HFD and CGA groups.

**Table 3:** Diversity and richness of gut microbiota in controls and chlorogenic acid‐treated groups of mice

Groups	Reads	OTUs	Chao1	Ace	Shannon	Simpson
ND	39,188 ± 6,399	2,637 ± 324	2,819 ± 632	2,868 ± 715	9.69 ± 0.08**	0.99 ± 0.007
HFD	48,674 ± 2,404	2,125 ± 469	2,481 ± 546	2,708 ± 602	8.51 ± 0.42	0.98 ± 0.024
CGA	41,461 ± 4,401	1,916 ± 201	2,312 ± 318	2,485 ± 351	8.27 ± 0.11	0.98 ± 0.010

*Notes*

Data indicate means ± *SD*.

HFD: high‐fat diet; ND: normal diet; CGA: chlorogenic acid.

**p <* *0.01 versus HFD group.

**Figure 3:**
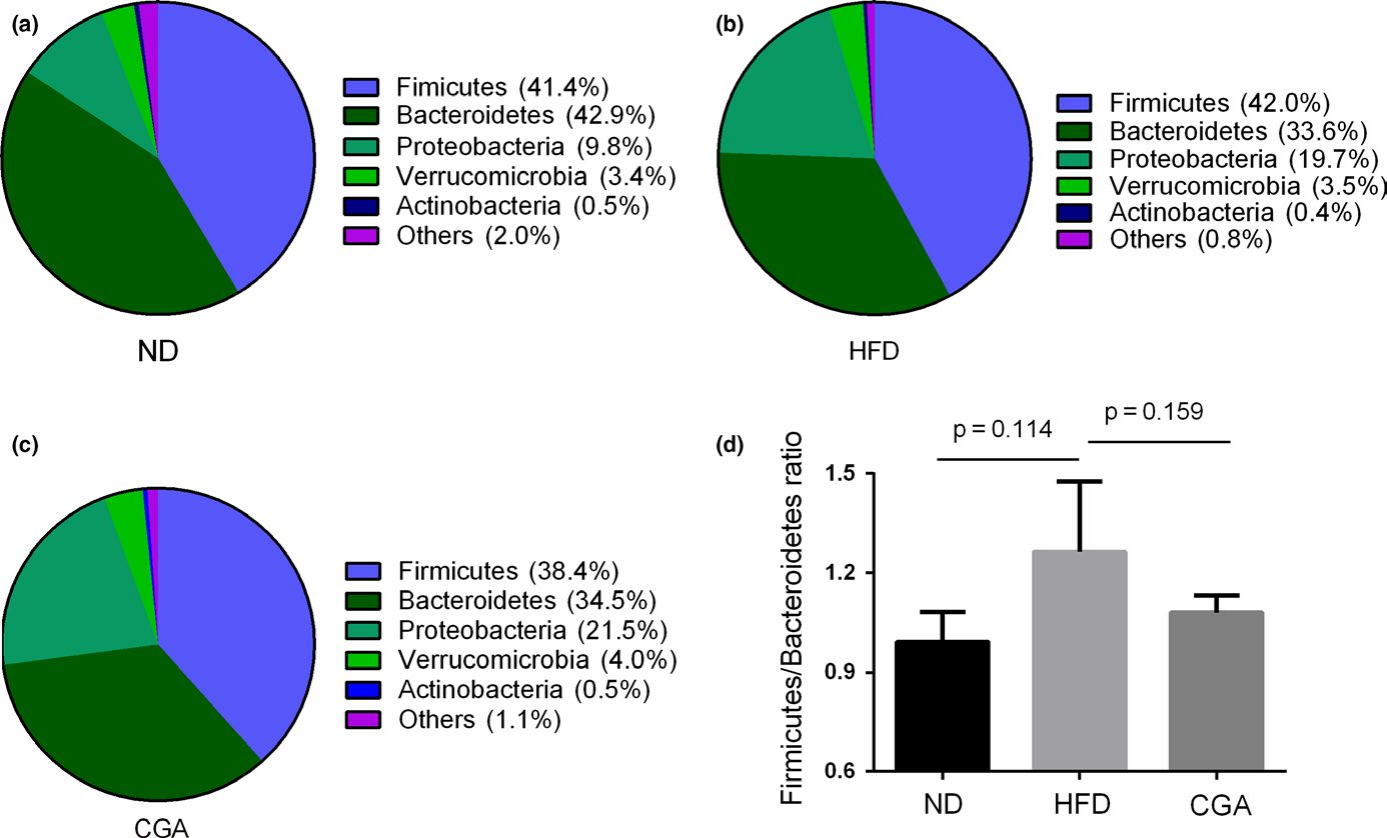
Distribution of the gut microbiota composition. ND group (a); HFD group (b); CGA group (c) at the phylum level and the ratio of *Firmicutes* to *Bacteroidetes* at different groups (d)

In addition, to profile the specific changes in the gut microbiota, the microbial community at the phylum level is shown in (Figure 3a–b). The most abundant phylum of *Firmicutes* accounting for 41.4% (in the ND group), 42.0% (in the HFD group), and 38.4% (in the CGA group) of the total bacterial sequences. The relative abundance of *Bacteroidetes* was 42.9% (in the ND group), 33.6% (in the HFD group), and 34.5% (in the CGA group). In addition, as shown in Figure 3d, the *Bacteroidetes*‐to‐*Firmicutes* ratio was modestly increased in the HFD group compared with that in the ND group. In contrast, after chronical administration chlorogenic acid for 6 weeks, a relatively lower *Firmicute*s: *Bacteroides* ratio was observed in the CGA group.

**Figure 4:**
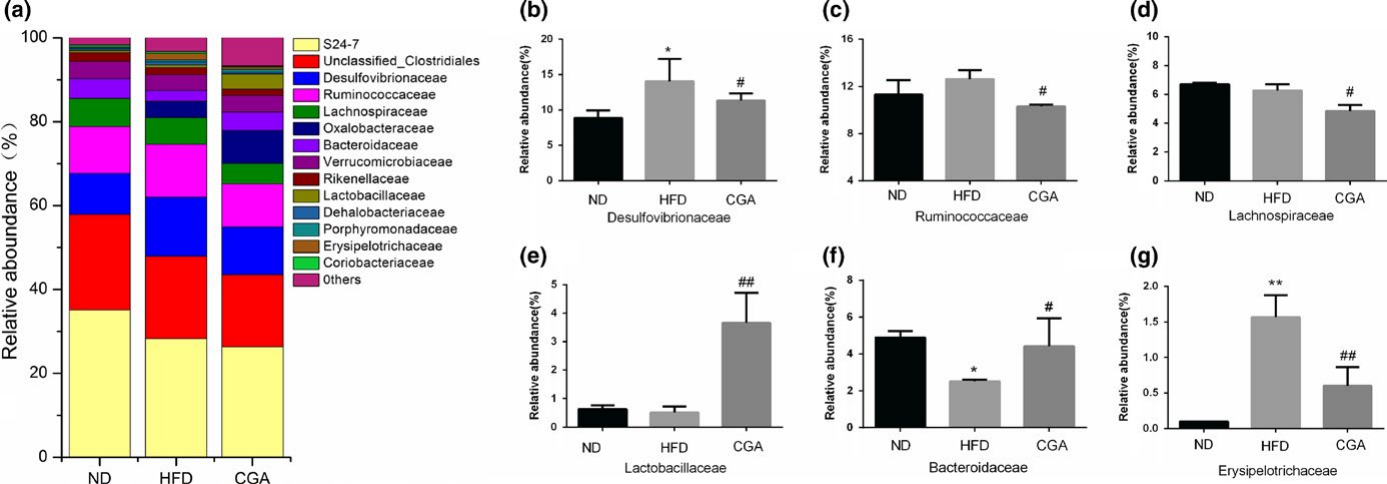
CGA modulated the gut microbiota composition at the family level. Family‐level taxonomic distributions of the microbial communities in cecum contents (a), The relative abundance of *Desulfovibrionaceae* (b), *Ruminococcaceae* (c), *Lachnospiraceae* (d), *Lactobacillaceae* (e), *Bacteroidaceae* (f) and *Erysipeiotrichaceae* (g) was expressed as the mean + *SD*. Significant differences between HFD versus ND are indicated: *p <* *0.05; **p <* *0.01. Significant differences between CGA versus HFD are indicated: ^#^ *p *<* *0.05; ^##^ *p *<* *0.01

At the family level, *S24‐7, Unclassified_Clostridiales,* and *Desulfovibrionaceae* accounted for a high proportion in three group*s* (Figure 4a). Moreover, the relative abundance of *Desulfovibrionaceae* in the HFD group was significantly higher than that in the ND group, but after chlorogenic acid treatment, the relative abundance of this bacteria decreased (Figure 4b). *Lachnospiraceae*, belongs to *Firmicutes* phylum, showed a slight drop in HFD group, but no significant difference compared with ND group (*p < *0.05), however, the flora community in chlorogenic acid group was decreased compared to that in the HFD group (Figure 4e). Additionally, incremental microbiota such as *Ruminococcaceae, Lactobacillaceae, Bacteroidaceaee,* and *Erysipelotrichaceae* were observed in the CGA group compared to HFD group (Figure 4c,d,f,g).

**Figure 5:**
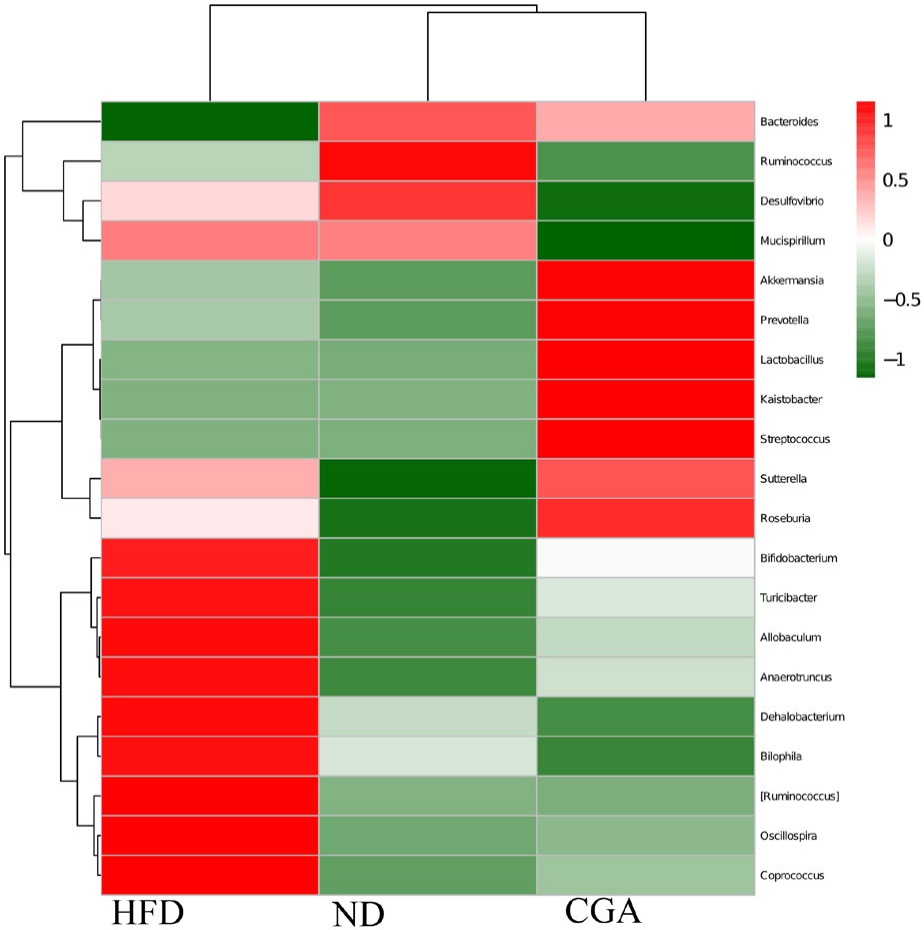
The heat map of 20 genera with the highest frequency and relative abundance in groups

The classification of the microbiota community structure at the genus level was assessed by a heat map (Figure 5). Apparently, genera were showed at different levels in three groups. Obviously, the relative abundance of *Oscillospira*,* Coprococcus*,* Anaerotruncus*,* Allobacterium, Bifidobacterium, Turicibacter* were increased, and a lower relative abundance of *Bacteroides* and *Ruminococcus* were exhibited in the HFD, but the changes of these species could be reversed by CGA treatment. Collectively, these results indicated that gut microbiota in HFD‐fed mice were modulated by chlorogenic acid.

## 4 DISCUSSION

In the previous study, chlorogenic acid has exhibited anti‐obesity property with improvements of lipid metabolism in HFD‐induced mice. Evidence suggests that chlorogenic acid (400 mg/kg) can achieve a deciline in TC, TG, and LDL‐C levels in plasma (Wu et al., 2014), in addition to this, chlorogenic acid (100 mg/kg) treatment also attenuated obesity‐related hepatic steatosis (Ma, Gao, & Liu, 2015). The present study confirmed this effect that chlorogenic acid (150 mg/kg) led to weight loss (*p < *0.05), suppressed lipogenesis, and ameliorated hepatic steatosis. There are evidences that obesity individuals are closely associated with higher TG, TC, LDL‐C levels, and lower HDL‐C level (Ko, Cockram, Woo, & Chan, 2001; Li, Huang, & Chen, 2008). Moreover, the declining ratio LDL‐C/HDL‐C, which is often considered to attenuate coronary heart disease risk related to obesity (Hwang et al., 2016). Here, it was revealed that chlorogenic acid could reverse plasma lipid changes altered by the HFD feeding, such as TG, TC, HDL‐C, LDL‐C, and LDL‐C/HDL‐C.

The adipose tissue is the most important organ for lipogenesis and metabolism of lipids and energy (Cariou et al., 2004). Here, we compared several mRNA expression of lipogenesis and lipolysis related genes in adipose tissue in mice received ND, HFD, or HFD+CGA, including PPAR‐γ, C/EBP‐α, SREBP‐1c (the major transcription factors in lipid regulation in vivo, Fève, 2005; Wanders, Graff, White, & Judd, 2013); AP2 (a protein expressed exclusively in differentiated adipocytes, correlated with cholesterol accumulation, Makowski, Brittingham, Reynolds, Suttles, & Hotamisligil, 2005); FAS and LPL (genes involved in regulation of fatty acid metabolism, and upregulation of this enzymatic activity is closely associated with obesity, Bull et al., 2002; Changsuk et al., 2010); PPARα and adiponectin (expression activation is known to facilitates the oxidation of fatty acid process, Faisal, Amin, & Sander, 2014; Karbowska & Kochan, 2006); GRP43 (which is highly expression in the white adipose tissue of HFD‐induced mice, Dewulf et al., 2011). In our study, CGA treatment markedly down‐regulated HFD‐induced PPAR‐γ, C/EBP‐α, SREBP‐1c, FAS, LPL, AP2, and GRP43 over‐expression in epididymal WAT. Meanwhile, CGA was also demonstrated to up‐regulated mRNA expression of PPARα and adiponectin, which were found to be down‐regulated by HFD. These changes in lipogenesis and lipolysis related genes may be also one of the underlying mechanisms by which CGA leads to low‐fat mass accumulation upon HFD challenge.

Obesity and related to metabolic disease are closed to the changes in gut microbial composition. Gut microbiota, particularly *Firmicutes and Bacteroidetes* are two major phyla in mice and human gut microbiota (Eckburg et al., 2005; Ley, Turnbaugh, Klein, & Gordon, 2006), and this phenomenon was also found in our study. Previous studies have suggested the obesity individuals owned a smaller number of *Bacteroidetes* and higher proportion of *Firmicutes* compared to lean individual (Rastmanesh, 2011; Turnbaugh, Backhed, Fulton, & Gordon, 2008). Here, the results showed that HFD‐induced mice had a relatively higher *Firmicutes/Bacteroidetes* ratio compared to the ND‐fed mice, but these could be inverted by administering chlorogenic acid.

However, other studies reported uncertain relationship between *Firmicutes/Bacteroidetes* ratio and obesity induction, in which overweight and obese individuals were found to have no variations in proportions of the ratio (Zhao, 2013) or have reduced *Firmicutes* and increased *Bacteroidetes* (Schwiertz et al., 2010). Increasing studies have pointed to a positive link between other bacterial phyla or special families and obesity (Table 4). In view of this, these species involved in energy metabolism also require further consideration, In this study, the increased relative abundance of *Ruminococcaceae* as well as its genus *Oscillospira* were found in HFD‐induced obesity mice, however, but CGA could significantly reverse the change of this species (*p *<* *0.05). *Lachnospiraceae*, a kind of digestive tract‐associated bacteria, correlates with increased fat mass and lipid level (Kameyama & Itoh, 2014; Murugesan et al., 2016; Pataky et al., 2016). However, in our study, a large decrease in *Lachnospiraceae* when HFD‐induced mice were simultaneously administrated chlorogenic acid. Meanwhile, our research also suggested chlorogenic acid promoted increase in the relative abundance of *Bacteroidaceae*,* and Lactobacillaceae* might prevent the negative metabolic phenotype correlated with obesity‐driven dysbiosis. It has been reported that a high abundance of *Erysipelotrichaceae* was observed in HFD‐induced mice, and they are strongly responsible for obesity (Hui et al., 2015). Interestingly, the relative abundance of *Erysipelotrichaceae* induced by high‐fat diet can be alleviated by CGA treatment. Intriguingly, the family *Desulfovibrionaceae* (*Proteobacteria* phyla) was thought to be positively associated with obesity (Delzenne & Cani, 2011), but a lower abundance was observed in the chlorogenic acid group, which may contribute to alleviating the development of obesity. Taken together, in line with the previous research that polyphenol‐induced intestinal microbiota homeostasis, we have reason to believe that chlorogenic acid shows anti‐obesity effect through beneficial modulation of the gut microbiota.

**Table 4:** The relationship of microbiota in family level as well as their genus and obesity in vivo studies

Phylum	Family	Host	Obesity‐specific measures	References
*Firmicutes*	*Ruminococcaceae* (+) *(Oscillospira* genus)	Humans (Obese)	Ester compounds (+)	Raman et al. (2013)
		Obese Patients	Visceral fat (+)	Pataky et al. (2016)
	*Lachnospiraceae* (+) (*Coprococcus* genus)	Mice (obesity)	Body fat (+)	Kameyama and Itoh (2014)
		Obese Patients	Total fat mass (+)	Pataky et al. (2016)
		Women (obesity)	BMI (+), Leptin levels (+)	Gomez‐Arango et al. (2016)
		Children (obesity)	Triglycerides level (+)	Murugesan et al. (2016)
	*Lactobacillaceae* (+) (*Lactobacillus* genus)	Broiler Chicks and Ducks	Weight gain (−)	Angelakis and Raoult (2010)
		MetS patients	Lipid level (−)	Isabel et al. (2016)
	*Erysipelotrichaceae* (+)		Cholesterol homeostasis (−), inflammation (+)	Kaakoush (2015)
		HFD‐induced mice	TC (+)	Hui et al. (2015)
*Bacteroidetes*	*Bacteroidaceae* (+) *(Bacteroides* genus*)*	Obese patients	Triglycerides level (−)	Isabel et al. (2016)
*Proteobacteria*	*Desulfovibrionaceae* (+)	Rat (obesity)	LPS (+)	Zhao, Zhang et al. (2017)

*Note*

(+) indicates an increase, (−) indicates a reduction.

HFD: high‐fat diet; TC: total cholesterol; LPS: lipopolysaccharides; MetS: metabolic syndrome.

## 5 CONCLUSIONS

The present study demonstrated that 6 weeks of chlorogenic acid administration could reduce the body weight, improve plasma lipid associated with HFD‐induced obesity and regulate lipogenesis and lipolysis genes expression in epididymal WAT. Moreover, chlorogenic acid treatment dramatically adjust the gut microbiota composition associated with obesity, such as decreasing *Ruminococcaceae, Desulfovibrionaceae, Lachnospiraceae, Erysipelotrichaceae,* and increasing *Bacteroidaceaea* and *Lactobacillaceae* with their genus members of the *Bacteriodes and Lactobacillus,* respectively. Our results demonstrated the potential possibility that chlorogenic acid in the prevention and treatment of obesity may closely rely on its role in regulation of gut microbiota.

## CONFLICT OF INTEREST

The authors declare no conflict of interests.

## ETHICAL APPROVAL

Animal experiment was conducted with the approval of the Experimental Animal Center of Fujian Medical University (Fu Jian, China) (Approval No.SYXK2016‐0006).
