# Supplementary material for: Chlorogenic acid alleviates obesity and modulates gut microbiota in high‐fat‐fed mice
Source: Food Sci Nutr. 2019 Jan 28;7(2):579–88. doi: 10.1002/fsn3.868 (PMC6392816; doi:10.1002/fsn3.868)
Supplement: Supplementary file 1 [file FSN3-7-579-s001.docx]

TABLE 1 Primer sequences for RT-PCR

| Target genes |  | Sequences | PCR product (bp) |
| --- | --- | --- | --- |
| β-actin | Forward | 5'–GTGCTATGTTGCTCTAGACTTCG-3' | 174 |
|  | Reverse | 5'–ATGCCACAGGATTCCATACC-3' |  |
| PPAR-γ | Forward | 5'–AGAACCTGCATCTCCACCTTAT-3' | 105 |
|  | Reverse | 5'-CCACAGACTCGGCACTCAAT-3' |  |
| PPAR-α | Forward | 5'-ATTTGCCAAGGCTATCCCA-3' | 125 |
|  | Reverse | 5'-GCATCCCGTCTTTGTTCATC-3' |  |
| FAS | Forward | 5'-ACCCTGACCCAGAATACCAAG-3' | 176 |
|  | Reverse | 5'-GTCAACAACCATAGGCGATTT-3' |  |
| LPL | Forward | 5'-CTGAGGATGGCAAGCAACAC-3' | 156 |
|  | Reverse | 5'-TGAGCAGTTCTCCGATGTCC-3' |  |
| C/EBP-α | Forward | 5'-GGCTCCTAATCCCTTGCTTTT-3' | 163 |
|  | Reverse | 5'-CTCTGTCTCCTACCACATGGCT-3' |  |
| AP2 | Forward | 5'-GATGCCTTTGTGGGAACCT-3' | 193 |
|  | Reverse | 5'-GTTTGAAGGAAATCTCGGTGTT-3' |  |
| SREBP-1c | Forward | 5'-ACAGACAAACTGCCCATCCA -3' | 223 |
|  | Reverse | 5'-GCAAGAAGCGGATGTAGTCG -3' |  |
| Adiponectin | Forward | 5'-ATCATTATGACGGCAGCACTG-3' | 104 |
|  | Reverse | 5'-GAGGCTCACCTTCACATCTTTC-3' |  |
| GPR43 | Forward | 5'-TGTTCAGTTCCCTCAATGCC-3' | 119 |
|  | Reverse | 5'-CAGCATAGAGGAGGCAGGATT-3' |  |
